# Are high nurse workload/staffing ratios associated with decreased survival in critically ill patients? A cohort study

**DOI:** 10.1186/s13613-017-0269-2

**Published:** 2017-05-02

**Authors:** Anna Lee, Yip Sing Leo Cheung, Gavin Matthew Joynt, Czarina Chi Hung Leung, Wai-Tat Wong, Charles David Gomersall

**Affiliations:** 1 Department of Anaesthesia and Intensive Care, The Chinese University of Hong Kong, 4th Floor, Main Clinical Block and Trauma Centre, Prince of Wales Hospital, Shatin, NT Hong Kong; 2 Intensive Care Unit, Prince of Wales Hospital, Shatin, Hong Kong

**Keywords:** Nurses, Personnel staffing, Workload, Critical care

## Abstract

**Background:**

Despite the central role of nurses in intensive care, a relationship between intensive care nurse workload/staffing ratios and survival has not been clearly established. We determined whether there is a threshold workload/staffing ratio above which the probability of hospital survival is reduced and then modeled the relationship between exposure to inadequate staffing at any stage of a patient’s ICU stay and risk-adjusted hospital survival.

**Methods:**

Retrospective analysis of prospectively collected data from a cohort of adult patients admitted to two multi-disciplinary Intensive Care Units was performed. The nursing workload [measured using the Therapeutic Intervention Scoring System (TISS-76)] for all patients in the ICU during each day to average number of bedside nurses per shift on that day (workload/nurse) ratio, severity of illness (using Acute Physiology and Chronic Health Evaluation III) and hospital survival were analysed using net-benefit regression methodology and logistic regression.

**Results:**

A total of 894 separate admissions, representing 845 patients, were analysed. Our analysis shows that there was a 95% probability that survival to hospital discharge was more likely to occur when the maximum workload-to-nurse ratio was <40 and a more than 95% chance that death was more likely to occur when the ratio was >52. Patients exposed to a high workload/nurse ratio (≥52) for ≥1 day during their ICU stay had lower risk-adjusted odds of survival to hospital discharge compared to patients never exposed to a high ratio (odds ratio 0.35, 95% CI 0.16–0.79).

**Conclusions:**

Exposing critically ill patients to high workload/staffing ratios is associated with a substantial reduction in the odds of survival.

**Electronic supplementary material:**

The online version of this article (doi:10.1186/s13613-017-0269-2) contains supplementary material, which is available to authorized users.

## Background

Staffing costs are the major contributor to the high cost of intensive care, with the major component being nursing staff. Although recent studies have demonstrated that decreased nurse/patient or nurse/bed ratios are associated with worse outcome [1–6], this finding is not universal [7–10] and the relationship between nurse workload-to-staffing ratios and patient outcome is unclear [11, 12]. This is reflected in the variability of nursing staff levels in different countries. In the UK, it is recommended nurse/patient ratios are at least one nurse to two patients [13]. In the USA, ratios range from 1.29 to 3.8 [10].

Most studies have examined the association between average nursing staff to patient ratios and outcome. However, the average nursing staff to patient ratio may be an insensitive measure. Firstly, it does not take into account case mix variability. In a study of 396 patients in one ICU, the daily Therapeutic Intervention Scoring System-28 (TISS-28) points varied from 13 to 58 [12]. Secondly, the use of average values implies that days of low nursing staffing can be compensated for by days of high staffing. This seems unlikely particularly as surveillance is a key role of intensive care nurses [10]: having two nurses detect an abnormality on one day will not compensate for an undetected abnormality on another.

A study examining the relationship between nursing shifts with and without a death found that a death was more likely when the nurse-to-patient ratio was lower, the patient turnover was higher, and the number of life-sustaining procedures was higher [14]. The number of life-sustaining procedures was used as a measure of workload, but this is not a validated measure of workload. Thus, the important question of whether the nurse workload/staffing ratio is associated with patient outcome remains unanswered.

Due to the complexities outlined above, the relationship between workload/staffing ratio and survival may be neither linear nor logistic. In particular, it is likely that there is a ceiling effect such that decreasing workload/staffing ratios improves survival, up to a certain point, and thereafter further decreases in workload/staffing have no effect. However, increasing workload/staffing ratios is less likely to be subject to a ceiling effect. Thus, a linear or logistic analysis is unlikely to be helpful. A more useful approach may be to dichotomize the workload/staffing ratios into understaffed and adequately staffed.

Unfortunately, there is no consensus on what constitutes understaffing. Although it has been suggested that an “accomplished” critical care nurse should be capable of managing 40–50 Therapeutic Intervention Scoring System-76 (TISS-76) points [15], this workload threshold has not been validated. Similarly, although 46 Nine Equivalents of Nursing Manpower Use Score (NEMS) points were assumed to be equivalent to the nursing activities of one nurse per day, the mean NEMS was only 26.5 in a large sample of ICUs [16]. However, if the threshold at which increasing staffing no longer produces improvements in survival can be identified, workload/staffing ratios above this can be defined as inadequate staffing and ratios below this, adequate staffing.

We hypothesized that exposure to inadequate workload/staffing ratio during a patient’s ICU stay is independently associated with increased mortality. We therefore carried out a cohort study to first identify a threshold workload/staffing ratio above which staffing was considered inadequate and then determined whether exposure to inadequate staffing at any stage of a patient’s ICU stay was associated with increased mortality after risk adjustment.

## Methods

### Design and participants

Approval to carry out the study was obtained from the Clinical Research Ethics Committee of The Chinese University of Hong Kong, which waived the need for patient consent. The study was a retrospective analysis of prospectively collected audit data from a cohort of consecutive patients admitted to two ICUs in Hong Kong over a period of 5 months. Patients were followed up until hospital discharge. Patients who had been admitted for <4 h, <16 years of age, with the diagnosis of burns or transferred to an ICU in another hospital were excluded.

### Setting

Both ICUs were the sole adult ICU in their respective hospitals, providing care for both medical and surgical patients. ICU1 was a 12-bed ICU in a 600-bed district hospital, while ICU2 was a 22-bed ICU in a 1400-bed tertiary referral university teaching hospital. Patients were largely cared for in open ward areas. Single rooms were only used for patients requiring isolation or when there were no other beds available. There were no student nurses, and nursing assistants’ role was restricted to turning, washing, applying pressure for haemostasis, collection of disposable equipment for procedures, cleaning of re-usable equipment and maintenance of stocks of disposable equipment. Typically, 2–3 assistants were rostered for every 10 patients. Respiratory therapists were not employed, but patients were assessed and treated by trained physiotherapists 1–2 times per day.

Nursing shifts were from 0700 to 1400, 1400 to 2100 and 2100 to 0700. Typically, night shifts were staffed with 75% of the number of staff on day shifts. There was no difference in staffing between weekdays and weekends. However, the two ICUs did not make day to day changes to their maximum patient capacity in response to the number of staff actually available (eg reduced numbers due to sickness), resulting in shift to shift variation in nurse/patient ratios. No non-regular staff were employed to make up any shortfall in nursing numbers. This gave us the opportunity to observe a natural experiment of the relationship between workload/staffing ratios and outcome.

In ICU1, the physician staffing during office hours consisted of a single on-site Intensivist supported by internal medicine residents. Night-time and weekend staffing consisted of internal medicine residents supervised by an Intensivist or Internal Medicine specialist, who was largely off-site but conducted daily ward rounds and was available to return to the ICU if required. In ICU2, 2–3 Intensivists provided on-site care during office hours. The exact number of Intensivists rostered was based purely on availability. A sole Intensivist provided on-site care from 0800 to 1500 at weekends. At all other times, a sole Intensivist was available to provide advice and, if necessary, to return to the ICU to provide direct patient care. At all times, the Intensivists were supported by intensive care and/or anaesthesia residents. If a doctor was unable to work, a replacement was found.

### Primary outcome

The primary outcome was survival to hospital discharge. This was collected from the electronic Hospital Authority clinical information system by one of the investigators (YSLC).

### Exposure

The exposure variable was the workload/staffing ratio threshold. Data required to calculate TISS-76 [15], Acute Physiology and Chronic Health Evaluation III (APACHE III) and the average number of bedside nurses working on each day were collected. TISS-76 data were collected by a dedicated audit nurse. For each day, the total TISS-76 and average total number of bedside nurses in the ICU were collected. A unit nursing workload/staffing ratio was calculated from the total TISS-76 divided by the total number of bedside nurses. The average number of nurses working on each day was calculated to allow for the fact that different shifts might have different numbers of nurses. A bedside nurse was classified as a nurse whose primary responsibility on that shift was direct patient care. Those nurses whose primary role was administrative or who were rostered to educational activities were excluded from the calculation. For each patient, the unit workload/staffing ratio that patient was exposed to was recorded each day during the patient’s ICU stay. Data on each individual nurse working in the ICU were also collected: years of ICU nurse experience and post-registration qualification in intensive care nursing.

### Statistical analysis

Data were checked for normality using Shapiro–Wilk’s test. Median and interquartile ranges are reported. The Mann–Whitney *U* test was performed to compare differences in medians between the two ICUs. The Chi-square test was used to test for differences in proportions. For the three patients with missing workload/staffing ratios, we assumed that these patients always had workload/staffing ratio <40. Pearson’s r was used to test for co-linearity between TISS and workload-to-staffing ratio.

The initial analysis was to determine the threshold workload/staffing ratio above which the probability of hospital survival is reduced. This, in effect, is the same as examining the incremental cost–effectiveness ratio of drug treatments (standard vs new) [17, 18]. We estimate the trade-off between extra ‘cost’ of workload (∆*W*) and extra ‘effect’ of staffing (∆*S*) on patient outcome. The comparison of workload and staffing in ratio form between survivors and nonsurvivors was transformed to linear values by using the net-benefit analysis framework methodology [17, 19]. Thus, the incremental net benefit (INB) was estimated by the following formula:

$${\text{INB}} = \left( {\Delta S \times \lambda } \right) - \Delta W$$

where *λ* is the maximum workload/staffing ratio threshold acceptable.

We used a series of mixed-effects regressions, with random intercepts by ICU location and patient, to estimate the INB. Using each patient’s net benefit (NB_*i*_) [defined as (*S* × *λ*) − *W*] as the dependent variable, we ran the following mixed-effect regression:

1 $${\text{NB}}_{i} = \beta_{0} + \beta_{\text{outcome}} {\text{Outcome}}_{i} + {\text{covariates}} + \varepsilon_{i}$$

where Outcome_*i*_ is the *i*th patient’s hospital discharge status (*β*_outcome_ = 1 for dead and 0 for alive) and *ε*_*i*_ is a stochastic error term. Equation 1 is fitted many times with different values of maximum workload/staffing ratio threshold acceptable (*λ*). The following covariates, included in the mixed-effects regressions, were selected for confounding adjustments on the basis of a causal directed acyclic graph (DAG) approach [20]: age, APACHE III score, readmissions, urgency of admission (elective or emergency), type of admission (medical or surgical), acute renal failure and ICU location (Additional file 1: Figure S1). We estimated the 90% confidence intervals (provides for 5%, one-sided test of hypotheses) around the INB from the regression results to determine the threshold at which decreasing workload/staffing ratio was associated with increased survival to hospital discharge and the threshold at which increasing workload/staffing ratio was associated with decreased survival. The analysis was at the patient-day level.

Once the thresholds were estimated, we internally validated our thresholds by performing multivariate logistic regression on a bootstrapped sample (1000 repetitions). The second part of the analysis was to model the relationship between a day or more of exposure to workload/staffing ratios above or below the identified threshold and changes in survival, adjusting for the same confounders as in the net-benefit regression analysis. An interaction between maximum workload-to-nurse ratio threshold and APACHE III score was included in the multivariate logistic regression models. We also included an intragroup correlation in the model to adjust for multiple admissions by the same patient.

Model calibration was assessed using the Hosmer–Lemeshow (HL) goodness-of-fit statistics with 8 degrees of freedom and plotting a calibration belt [21]. The calibration belt is a fitted polynomial logistic function curve between the logit transformation of the predicted probability and outcome with surrounding 80% CI (light grey area) and 95% CI (dark grey area) [21]. The calibration belt is more useful than the HL test as it highlights ranges of significant miscalibration [21]. To assess the discrimination performance, an area under the receiver operating characteristic (AUROC) curve was constructed and a c-statistic was estimated. The Nagelkerke’s *R* ^2^ was used to estimate the overall performance of the logistic regression model. Statistical analysis was performed using STATA version 14 (StataCorp, College Station, TX) and the calibration belt was plotted using R version 3.2.5 (R Foundation for Statistical Computing, Vienna, Austria).

## Results

There were 925 admissions during the study period. Thirty-one were excluded: 11 admitted for less than 4 h; 9 transferred to an ICU in another hospital; 4 aged less than 16 years, and 7 had burns as the primary diagnosis. Thus, there were 894 separate admissions in the cohort (Table 1), representing 845 patients. Among the 894 episodes, there were 98 deaths in ICU and 166 deaths before hospital discharge. There were 242 and 652 episodes in ICU1 and ICU2, respectively. Characteristics of patients by ICU are shown in Table 1 and at hospital discharge in Table 2. Nurses in ICU1 had a median of 2 (IQR 1–5) years intensive care nursing experience. Those in ICU2 had a median of 5 (IQR 4–7) years of experience (*P* < 0.001). In ICU1, 28% and in ICU2 67% of nurses had a post-registration qualification in intensive care nursing (*P* < 0.001). This qualification involves an additional year of part-time training, placement in a training ICU, coursework and formal examination.

**Table 1:** Characteristics of patients by Intensive Care Unit (ICU)

	ICU1 (242 episodes)	ICU2 (652 episodes)	*P* value
Median (IQR) age (years)	69 (55–76)	61 (46–72)	<0.001
Number of males/females	171:71	371:281	<0.001
Median (IQR) APACHE III	62 (44–83)	54 (38–77)	0.001
Number of elective ICU admissions (%)	99 (41)	225 (35)	0.08
Number of patients with acute renal failure (%)	13 (5)	30 (5)	0.63
Number of surgical patients (%)	111 (46)	404 (62)	<0.001
Number of cardiac surgical patients (%)	0 (0)	53 (8)	<0.001
Median (IQR) total TISS-76 score/episode	86 (52–166)	79 (51–139)	0.29
Median (IQR) bedside nurses per patient per day during ICU stay	2.6 (1.5–4.9)	2.0 (1.3–3.7)	<0.001
Median (IQR) total TISS/nurse ratio	36 (30–39)	40 (36–43)	<0.001
Number of patients with more than one ICU admission (%)	11 (5)	38 (6)	0.45
Median (IQR) length of stay in ICU	4 (2–6)	2 (2–5)	<0.001
Number of in-ICU deaths (%)	25 (10)	73 (11)	0.71
Number of in-hospital deaths (%)	44 (18)	122 (19)	0.86
Median (IQR) APACHE III-predicted risk of death (%)	15 (5–34)	8 (2–30)	0.001

*IQR* interquartile range, *APACHE III* Acute Physiology and Chronic Health Evaluation III, *TISS-76* 76 item Therapeutic Intervention Scoring System

**Table 2:** Characteristics of patients at hospital discharge

	Mortality (166 episodes)	Survival (728 episodes)	*P* value
Median (IQR) age (years)	69 (52–76)	62 (46–72)	0.001
Number of males/females	99:67	443:285	0.77
Median (IQR) APACHE III	92 (73–119)	50 (36–68)	<0.001
Number of elective ICU admissions (%)	22 (13)	302 (41)	<0.001
Number of patients with acute renal failure (%)	33 (20)	10 (1)	<0.001
Number of surgical patients (%)	62 (37)	453 (62)	<0.001
Number of cardiac surgical patients (%)	3 (2)	50 (7)	0.01
Median (IQR) total TISS-76 score/episode	152 (86–274)	74 (48–120)	<0.001
Median (IQR) bedside nurses per patient per day during ICU stay	4.1 (2.3–7.6)	1.9 (1.2–3.2)	<0.001
Median (IQR) total TISS/nurse ratio	39 (35–41)	39 (35–42)	0.77
Number of patients with more than one ICU admission (%)	11 (7)	38 (5)	0.47
Median (IQR) length of stay in ICU	3 (1–7)	2 (1–4)	<0.001
Number of in-ICU deaths (%)	98 (59)	0 (0)	<0.001
Median (IQR) APACHE III-predicted risk of death (%)	54 (28–81)	6 (2–19)	<0.001

*IQR* interquartile range, *APACHE III* Acute Physiology and Chronic Health Evaluation III, *TISS-76* 76 item Therapeutic Intervention Scoring System

**Fig. 1:**
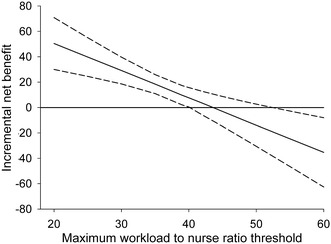
Maximum workload/nurse ratio with 90% confidence intervals from the net-benefit regression analysis

The results of the mixed-effects regression analysis to identify threshold values are shown in Fig. 1. The lower 90% confidence interval crosses zero when the workload/staffing ratio is 40. This indicates that there is more than 95% probability that survival to hospital discharge is more likely to occur when the maximum workload-to-nurse ratio is <40. The upper 90% confidence interval crosses zero when the ratio is 52, indicating that there is a more than 95% chance that death is more likely to occur when the ratio is >52.

**Fig. 2:**
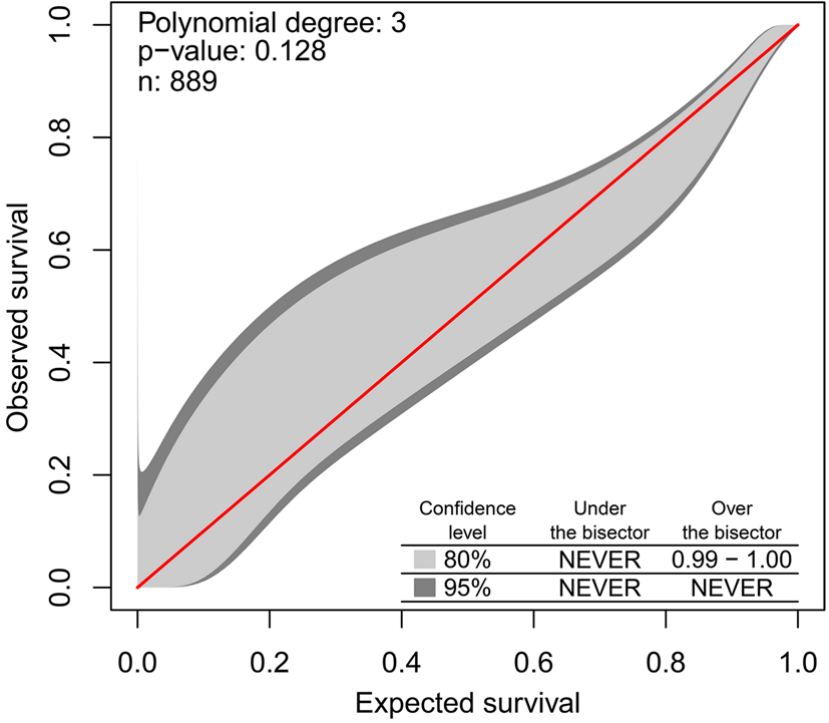
Calibration belt for logistic regression model examining the effect of workload/staffing ratio threshold set at 40 on survival

There were 275 admissions with workload/staffing ratio always <40 during the ICU stay. In the model examining the relationship between survival and workload/staffing in these patients, there was a significant interaction between workload/staffing ratio and APACHE III score (*P* < 0.01). The calibration belt was acceptable (Fig. 2) despite a significant HL test (*P* = 0.002), and the AUROC was 0.88 (95% CI 0.85–0.90). The overall performance of the model was satisfactory (*R* ^2^ = 0.45). Among patients with an APACHE III score of 60, patients with workload/staffing ratio of always <40 during the ICU stay were twice as likely to survive to hospital discharge (odds ratio 2.28, 95% CI 1.07–4.80) than other patients with same severity of illness but receiving higher workload/staffing ratio (ie. sometimes or always above 40). When APACHE III was between 70 and 130, a workload/staffing ratio always less than 40 was not significantly associated with survival. Survival was less likely to occur when APACHE III score was above 130 even when workload/staffing ratio was always less than 40 (odds ratio 0.24, 95% CI 0.09–1.01 at APACHE III score = 130). Crude mortality was 10.5% (ICU mortality) and 15.6% (hospital mortality) when workload/staffing ratio was always less than 40 during the patients’ ICU stay.

There were 27 admissions with workload/staffing ratio of ≥52 during the ICU stay (10 admissions had workload/staffing ratio of ≥52 for 50–100% of the time). In ICU2, there was one day when the workload/staffing ratio was 55. On the two days when workload/staffing ratio was ≥52, which occurred in ICU2 (22 beds), the mean number of nurses was 17 and the mean total unit TISS was 911. In the model examining the relationship between survival and workload/staffing ratio at 52, there was no significant interaction between workload/staffing ratio and APACHE III score (*P* = 0.94), and thus the final model without an interaction term was selected. The calibration was acceptable [HL test *P* = 0.21, calibration belt showed no over- or under-prediction intervals (not shown)] and the AUROC was 0.88 (95% CI 0.85–0.90). The overall performance of the model was satisfactory (*R* ^*2*^ = 0.44). After adjusting for confounders, patients with high workload/staffing ratio (≥52) for one or more days during their ICU stay were less likely to survive to hospital discharge (OR 0.35, 95% CI 0.15–0.84) than other patients with lower workload/staffing ratio (always <52). Crude mortality was 18.5% (ICU mortality) and 29.6% (hospital mortality) when workload/staffing ratio was ≥52 for at least one day during the patients’ ICU stay.

There was no correlation between average patient TISS and workload/nurse ratio (*R* ^2^ = 0.04).

## Discussion

Our data demonstrate that there is an association between nurse workload/staffing ratios and hospital survival, above certain thresholds and depending on severity of illness. In our setting, a TISS per nurse of 52 or more was associated with an adjusted odds ratio of 0.35 for survival. This applied to all patients regardless of severity of illness. A TISS per nurse of <40 for the patient’s entire ICU stay was not associated with an increased chance of survival for extremely sick patients but was associated with increased survival among those who were only moderately severely ill (APACHE III ≤60). Our finding that a threshold of 52 TISS points was the workload/staffing ratio above which all patients (in our two ICUs) had an increased risk of death corresponds closely to the previous suggestion that a highly “accomplished” critical care nurse should be capable of managing up to 50 TISS points [15]. This relationship between workload/staffing ratios and outcome may explain our finding that both ICUs had similar crude mortalities despite the fact that the severity of illness, measured by APACHE III, was higher in ICU1. In ICU1 the median TISS/nurse ratio was significantly lower than in ICU2 (36 vs 40) (Table 1).

TISS-76 is a revised form of the original TISS score and uses 76 possible interventions to quantify nursing workload [15]. To give an example of the workload which corresponds to 52 TISS points, a paralysed mechanically ventilated patient who required emergency surgery in the past 24 h, receiving more than one vasoactive drug, amiodarone infusion, parenteral nutrition, renal replacement therapy, non-scheduled bolus and intermittent scheduled IV medication, two IV antibiotics, endotracheal suction, 6 units of blood or fresh frozen plasma transfusion in 24 h, platelet transfusion, multiple stat blood tests, hourly neurological and non-neurological observations, fluid input and output monitoring, electrocardiographic monitoring, with an arterial line, central venous catheter, nasogastric tube and urinary catheter in situ would score 52 points.

Two other studies of workload/staffing ratios have quantified workload in terms of nursing interventions rather than number of patients. Castillo-Lorente et al. [22] were unable to demonstrate a statistically significant increase in ICU mortality with increasing peak TISS-28-to-nurse ratios. However, the TISS-28-to-nurse ratio in the high workload/staffing group was >29.8 TISS-28 points per nurse [22]. TISS-28 is a simplified version of TISS-76 which was devised, in part, to reduce the time required for data collection [23]. Although TISS-28 and TISS-76 are not directly comparable, a previous study suggests that a TISS-28 of 29.8 corresponds to a TISS-76 of approximately 30 [22]. This is well below our lower threshold of 40, although for reasons discussed below the absolute values of these thresholds should be interpreted with caution. Neuraz et al. demonstrated that one or more death on a shift was associated with a patient-to-nurse ratio >2.5 and a greater number of life-supporting procedures per patient [14]. Although it is difficult to directly compare these data with ours, the threshold demonstrated by Neuraz et al. [14] appears high, if 50 TISS points are achievable by an accomplished critical care nurse. However, this may simply reflect the nature of their analysis. For the patient-to-nurse ratio to have an impact on mortality on the same shift would likely require a very major deficit in nursing care.

Our workload/staffing ratio was calculated on a unit basis rather than individual patient basis because this more closely reflects the way our nursing teams work. Our patients are largely nursed in open ward areas, and while there is one nurse with primary responsibility for each patient, the work is shared between the nurses. The choice of TISS score rather than newer scores such as the Nursing Activities Score [24] also reflects the work carried out by our nurses and the way we measured staffing. We measured staffing by counting the number of nurses involved in direct patient care, excluding those with predominantly managerial and administrative tasks. We also did not count the number of healthcare assistants (nurse assistants). Healthcare assistants are mainly responsible for carrying out tasks like washing patients, changing linen, special room cleansing procedures and patient positioning. Thus several of the additional tasks added to TISS to create the Nursing Activities Score [24] are not carried out by our bedside nurses.

Calculating workload/staffing ratios on a unit basis also minimized the risk of confounding by severity of illness. Greater severity of illness is associated with increased mortality and increased TISS for an individual patient. However, a greater severity of illness of an individual patient is unlikely to substantially alter the unit TISS/nurse ratio in a 22 bedded unit. This is supported by the absence of a correlation (*R* ^2^ = 0.04) between patient TISS and unit TISS/staffing ratio (our measure of workload/staffing). This indicates that our finding that workload/staffing ratio is associated with hospital survival is unlikely to be due to confounding by severity of illness.

The magnitude of adjusted odds ratio and the fact that the duration of exposure to high workload/staffing ratios associated with poor outcome may have been as little as one day, should raise questions about the advisability of “making do” when there is a shortage of staff. While it can be argued that it is inappropriate to deny ICU admission to a patient when there are available bed spaces, it is important to recognize that this may have a significant impact on survival for those patients already in the ICU if staffing levels are inadequate. Furthermore, it should raise questions about whether ICUs should be staffed on the basis of actual workload rather than number of patients [12] and conversely, whether bed capacity should be determined by actual nursing workload and staffing rather than physically empty beds, and about plans for sustainable ICU expansion in the face of disasters. The recent ACCP statement on surge capacity in a disaster recommended that ICU capacity be increased by up to 200% above baseline capacity [25]. Our data suggest that if the physical bed capacity is not matched by an increase in staffing, there may be a very substantial decrease in survival, which will at least partially negate any beneficial effect of increasing beds.

As our study was a purely observational study, it cannot demonstrate a causal relationship between workload/staffing ratios and hospital survival. Despite our use of risk adjustment techniques, there remains a possibility of residual confounding. However, there are other studies which support our findings [1–3, 10] and there are plausible mechanisms by which increased workload/staffing ratios might decrease survival. These mechanisms include the association of lower ICU nurse staffing levels with increased morbidity such as prolonged duration of weaning [26], increased nosocomial infection [2, 27–31], inadequate nutrition [32], pressure sores [2] and critical incidents [33].

Our study has a number of other weaknesses. Firstly, the analysis was retrospective. However, the data were prospectively collected. Secondly, the data were collected in only two ICUs. This may limit the generalizability of the results. Although our thresholds are considerably higher than those used by Castillo-Lorente et al. [22], we believe that the workload/staffing ratios in our ICUs are not unusual. Altafin et al. [34] reported a mean TISS 28 score that equates to a TISS 76 of 24.5 with a nurse-to-patient ratio of 1:10 and a nursing technician ratio of 1:2. Even counting the technicians as nurses, this would give a mean ratio of close to 50. Blatnik [35] reported a mean TISS-28/nurse ratio of 47, equating to a TISS-76/nurse ratio of 54. Even in high-income countries, workload/staffing ratios may be high, exceeding the equivalent of a TISS-76/staff ratio of 64 on 3% of all patient days in an ICU in the Netherlands [36]. Indirect data also support our contention, TISS-28 scores equivalent to a TISS-76 score of 35–48 [35, 37, 38] were reported from Germany, Slovenia and Columbia. Unless nurse/patient ratios (which were not reported) were greater than 1:1, this would suggest a substantial proportion of patients were exposed to the levels of staffing that are associated with worse outcome. Survey data suggest the mean nurse/patient ratio in Germany is 1:2.7 [39]. Nevertheless, we would caution against the direct application of our specific thresholds of 40 and 52 in determining how many nurses other individual ICUs require. Levels of training and the roles carried out by nurses differ in different ICUs. In particular, the role and number of supporting staff may affect the appropriate nurse workload/staffing ratio for a particular ICU. Our ICUs consisted predominantly of open areas with few individual rooms. Although this may be unusual in some parts of the world, it is common in Asia where only 36.9% of ICU beds are in individual rooms and 14.5% of ICUs have no single rooms [40]. Thirdly, we did not record limitation of therapy orders. It is possible that limitation of therapy may have resulted in some confounding by increasing the risk of death while reducing measured nursing workload. This would have the effect of reducing the probability of finding an association between high workload/staffing ratios and survival, making our finding more robust. Furthermore, any such effect would have been minimized by our method of calculating workload/staffing ratios. We calculated daily unit workload/staffing ratios and then assigned that ratio to each patient present in the ICU on that day rather than calculating daily individual patient workload/staffing ratios.

Ultimately, we believe that the optimal design to establish a causal relationship between nurse workload/staffing ratios and mortality is a cluster randomized controlled trial or a multi-centre step-wedge interrupted time series to compare the effect of determining bed capacity based on workload/staffing ratios against bed capacity determined by physical bed spaces. However, considerable preparatory work will be required before such studies can be properly designed. The first step is to confirm our findings prospectively.

## Conclusions

Our data indicate that exposure to as little as one day of high workload/staffing ratios is associated with a substantially increased risk of death in critically ill patients. This confirms a previous finding that excessive workload/staffing ratios are associated with increased mortality [14] and refutes the findings of a study involving relatively low workload/staffing ratios that suggested there is no relationship between workload/staffing ratios and outcome [22]. If confirmed, our finding has significant implications for nurse staffing in Intensive Care Units, suggesting that staffing should be based on workload, not just patient numbers, and that “making do” with fewer nurses even for a short time or temporary increases in ICU capacity without a corresponding increase in staffing may adversely affect patient outcome.

## Additional file

**Additional file 1: Figure S1.** Directed acyclic graph. Assumptions made in the workload/staffing ratio threshold and hospital mortality relationship to identify the set of variables needed for confounding adjustment.

## Abbreviations

ACCP: American College of Chest Physicians
APACHE: Acute Physiology and Chronic Health Evaluation
AUROC: area under the receiver operating characteristic
CI: confidence interval
DAG: directed acyclic graph
HL: Hosmer–Lemeshow
ICU: Intensive Care Unit
INB: incremental net benefit
IQR: interquartile range
NEMS: Nine Equivalents of Nursing Manpower Use Score
OR: odds ratio
TISS: Therapeutic Intervention Scoring System
