# Supplementary figures and images for: Are high nurse workload/staffing ratios associated with decreased survival in critically ill patients? A cohort study
Source: Ann Intensive Care. 2017 May 2;7:46. doi: 10.1186/s13613-017-0269-2 (PMC5413463; doi:10.1186/s13613-017-0269-2)

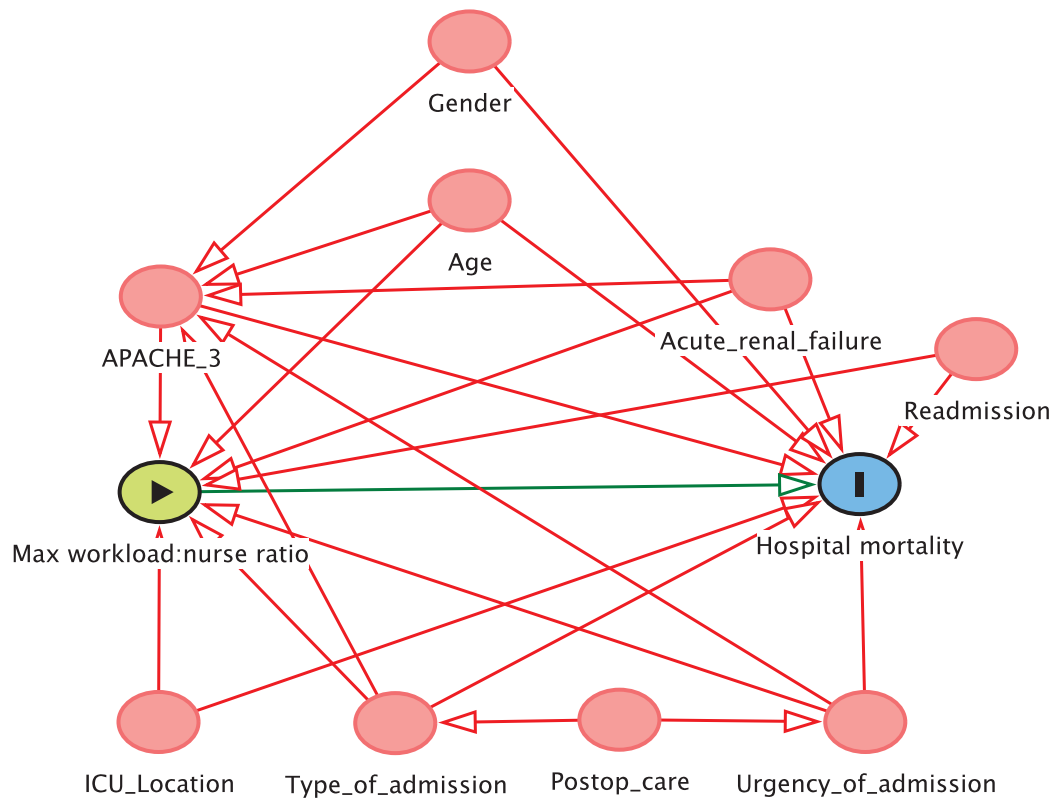

Supplement: Supplementary file 1 — Additional file 1: Figure S1. Directed acyclic graph. Assumptions made in the workload/staffing ratio threshold and hospital mortality relationship to identify the set of variables needed for confounding adjustment. [file 13613_2017_269_MOESM1_ESM.pdf]
